# ‘The grave must not be seen by anyone!’: Beliefs and practices about stillbirths in Eastern Uganda

**DOI:** 10.1186/s12978-025-02022-0

**Published:** 2025-04-29

**Authors:** Martin Chebet, Joseph Rujumba, Kathy Burgoine, David Mukunya, Noela Regina Akwi Okalany, Peter Olupot-Olupot, Thorkild Tylleskär, Andrew D. Weeks, Agnes Napyo, Ingunn Marie S. Engebretsen

**Affiliations:** 1 Department of Paediatrics and Child Health, Busitema University Faculty of Health Sciences, Mbale, Uganda; 2 Centre for International Health, Department of Global Public Health and Primary Care, University of Bergen, Bergen, Norway; 3 Department of Paediatrics and Child Health, Makerere University College of Health Sciences, Kampala, Uganda; 4 Mbale Regional Referral Hospital, Mbale, Uganda; 5 Department of Community and Public Health, Busitema University Faculty of Health Sciences, Mbale, Uganda; 6 Mbale Clinical Research Institute, Mbale, Uganda; 7 Sanyu Research Unit, Department of Women’s and Children’s Health, University of Liverpool, Liverpool, UK; 8 Department of Nursing, Kabale University, Kabale, Uganda

**Keywords:** Stillbirths, Beliefs, Practices, Eastern Uganda, Culture, Hidden, Stigma

## Abstract

**Background:**

Nearly half of all stillbirths occur in sub-Saharan Africa and accurate registration could inform reduction efforts. We explored the beliefs and practices surrounding stillbirths in Eastern Uganda, revealing cultural factors that could influence the accurate counting of stillbirths.

**Methods:**

We conducted a qualitative study among women with a history of stillbirths, and men, women and community leaders with an experience of childbirth in Eastern Uganda. The study also included healthcare workers from three health facilities. We conducted 30 in-depth interviews and six focus group discussions to explore the beliefs and practices about stillbirths. All discussions and interviews were audio recorded and transcribed into English. Thematic analysis was done using NVivo R1 (2020) software for coding.

**Results:**

We enrolled a total of 74 participants: 44 in six focus group discussions and 30 in in-depth interviews. Four themes emerged: first, the community believed that stillborn babies can be used for witchcraft or as a source of curses therefore stillbirths were hidden from the public. Second, women were useful in marriage only when they bore live children and were despised when they had a stillbirth leading to discord in marriage and stigma. Third, stillborn babies were not considered human and therefore, the baby was not named or buried in a coffin. Fourth, the spirit of the stillborn baby was considered harmful to the next siblings and their parents sought the services of traditional healers and witch doctors to protect these siblings.

**Conclusion:**

The immediate tragedy of a stillbirth has long-term personal and societal effects on the mother, resulting in stigma, marital breakup and isolation. The secrecy about stillbirths may also contribute to underreporting of stillbirths. Efforts to improve documentation of stillbirths and support for families who have had stillbirths need to incorporate culturally sensitive interventions.

## Background

Globally, about 2 million stillbirths occur annually, most of which occur in low- and middle-income countries (LMICs) [1]. Worldwide, the stillbirth incidence decreased from 21.3 to 13.9 per 1000 total births between 2000 and 2021. Although gains have been made, stillbirth rates remain high in many LMICs [2]. Uganda’s stillbirth rate is estimated to be 15.2 per 1000 total births [3].

To reduce the global burden of stillbirths, it is crucial to determine the magnitude of the problem through accurate recording and reporting. Studies have, however, shown that communities in some LMICs have beliefs and practices about stillbirths that may significantly affect the recording and reporting [4–7]. Stillbirth rates are potentially underestimated in LMICs because they are often from a national registry based on hospital data [8]. Most of the published studies on the incidence of stillbirth in Uganda are from retrospective reviews, population-based studies and facility-based registers [9–12].

In Uganda, studies have shown varying influence of culture on the practices about stillbirths [4, 5, 13]. It is important to understand the type, extent and reasons for these practices from different Ugandan communities to guide designing and implementing tailored national programmes to improve the accuracy of the reported burden of stillbirths. In addition, experiences of health workers around stillbirths should also be explored to inform any future public health programmes targeting stillbirths. In this study, we aimed to describe the beliefs and practices surrounding stillbirths in Mbale, Eastern Uganda.

## Subjects and methods

### Study design

We conducted a qualitative descriptive study between October 2023 and August 2024 in Eastern Uganda.

### Study setting

The study was conducted among community residents of Mbale District and Mbale City in Eastern Uganda. Mbale City is the main municipal, administrative and commercial centre of Mbale District. The formal boundaries of Mbale City extend beyond the urban area, so most participants residing in Mbale City, lived in predominantly rural areas. Mbale is one of the six districts occupied by the Bagisu, Gisu, Gishu or Bamasaba, one of the major ethnic groups in Uganda with an estimated population of two million. It is a predominantly rural area where most of the population have not gone beyond primary school. Although Lumasaba is the language spoken by these people, Luganda is also widely spoken especially in peri-urban and urban areas within or close to Mbale City. It is a paternalistic society where marriage is usually validated only after payment of the bride price by the groom to the parents of the bride. A husband is considered to have authority over his wife after the payment of the bride price. If the wife leaves the marriage, her parents must pay back the bride price. Polygamy is a common practice in this community and children are so highly regarded in marriage that if a wife does not bear live children, the husband may dissolve the marriage or marry a second wife.

The study was also done among health workers at Mbale Regional Referral Hospital (MRRH), Busiu Health Centre IV and Nakaloke Health Centre III.

### Participants

The participants included women and men aged above 20 years who had previously given birth or whose spouse had previously given birth and who were residents of the selected communities; women with a history of stillbirth; community health workers (CHWs); and religious, cultural and community leaders. The age of 20 years was chosen to increase the likelihood of participants having had personal experiences of stillbirth practices in this community. The study population also included healthcare workers (midwives and doctors) who had had at least one year of experience working in a labour ward, postnatal ward or antenatal clinic at Mbale RRH, Busiu Health Centre IV and Nakaloke Health Centre III.

We purposively selected participants from within a10-kilometre radius of Busiu and Nakaloke Health Centres. The CHWs, community leaders and religious leaders were purposively selected with the help of the sub-county leaders. Community residents and women with a history of a stillbirth were purposively selected with the help of the CHWs.

The nurse or doctor in-charge of each facility was informed about the target participant number, the cadres and the experience of staff required in their health facility. They then provided the names and contact details of the selected healthcare workers. If the healthcare worker was unavailable, the nurse/doctor in charge was asked to provide an alternative name. Only one selected healthcare worker did not participate in the study because of annual leave.

### Data collection

In-depth interviews (IDIs) and focus group discussions (FGDs) were conducted by the lead author (MC), a paediatrician, together with four trained research assistants who were graduate midwives with previous experience in conducting qualitative research. The IDIs were with healthcare workers, cultural leaders, religious leaders and women who had experienced a stillbirth or a close relative with such an experience.

The FGDs included women and men living in the study area with experience of childbirth and this included different population strata in terms of sex and age. We conducted six FGDs of 6–8 participants including: women aged between 20 and 35 years; women aged above 35 years; men aged between 20 and 45 years; men aged more than 45 years; CHWs; and Muslim participants only. Religion was not an inclusion or exclusion criteria but a group comprising of only Muslim participants only was included based on recommendations arising from data analysis that suggested they may hold unique beliefs and practices around stillbirths. At the beginning of the study, FGDs and community-based IDIs were conducted concurrently but the final community-based IDIs were performed in parallel with the IDIs with healthcare workers.

Each IDI was conducted in a language that the individual participant was fluent in. Most community participants were fluent in Lumasaba or Luganda, while the healthcare workers were all fluent in English. The language of the IDI was agreed upon between the interviewer and participant before the day of the interview. The majority of IDIs were done in Lumasaba and a few were in Luganda or English. Four FGDs were conducted in Lumasaba and two (FGDs for CHWs and Muslims) in Luganda. The two interviews conducted in Luganda each had about half of the participants residing in a peri-urban environment in Mbale City. The rest of the participants were from the rural areas. All the interviewers were fluent in English, two in Lumasaba and three in Luganda. 

The participants were approached to make an appointment for an interview either through a telephone call or in person. An appropriate venue was agreed upon between the researcher and the participant. All the FGDs were conducted in a health facility; about half of the IDIs were conducted at a health facility; and the remaining IDIs were conducted in the community. The IDIs with healthcare workers were done in their places of work. The interview room contained only the researchers and the participants; was quiet and free from disruptions; and allowed for confidentiality.

Each FGD began by the research assistant providing general information about stillbirths. The participants were then asked to share their individual experiences about stillbirths moderated by MC or an appointed research assistant fluent in the language in which the interview was being conducted. Each FGD was done using an interview guide collectively created by the authors and translated into the local languages. It was piloted to ensure that the questions elicited the responses the authors intended to capture. The interview guide asked participants about their experience of stillbirths and the common beliefs and practices in the community. The interview guide also included probes about practices during and after the funeral ceremony. Each FGD lasted 90–120 min while the IDIs lasted between 30–60 min. Field notes were taken to capture cues and non-verbal communication that were not possible to capture through an audio recording. No repeat interviews were done. Data were collected until there was no additional information arising from the data. The authors did not return the transcripts to the participants for comments because it was not logistically possible.

### Data management and analysis

The interviews were audio recorded and then transcribed verbatim. The transcripts in the local languages were translated to English. Data were de-identified and stored electronically in password protected files. The paper copies of documents with identifiable information for participants were stored under lock and key.

The coding and analysis were done with NVivo R1 (2020). Thematic content analysis was done following the steps described by Braun and Clarke [13]. To familiarise ourselves with the data, we read and re-read the transcripts. Codes were then generated by attaching meaning to different statements made in the data by reading few transcripts. The analytical process involved theme generation which was an iterative process merging similar codes or breaking groups of codes to subthemes and themes. Coding and categorisation were done by MC and AN and cross checked by the rest of the authors. We ensured that the themes matched the coded extracts and the entire data set. The subsequent transcripts were then coded using the existing categories and codes. Finally, interpretation of the data was done and extracts made from the analysed data to write the manuscript.

### Validation meeting

To verify that the results reflected true beliefs and practices of the community studied, we organised a validation meeting with 11 individuals who had not participated in the earlier data collection. This team consisted of four elders (two women and two men), two CHWs, two healthcare workers, two religious leaders and one community leader. We presented the results of the study to them and asked them whether these beliefs and practices existed in their communities, how common they were and the reasons behind them.

### Ethical considerations

The study was done in accordance with the Declaration of Helsinki [14]. Ethical approval was obtained from Uganda National Council of Science and Technology (reference number HS3112ES) and the local ethics committee (Busitema University Faculty of Health Sciences Research and Ethics Committee, reference number BUFHS-2022–18) and Regional Committee for Medical and Health Research Ethics, Norway (REK West #278,845). Written informed consent was obtained from each participant before enrolment. The midwives provided individual confidential counselling for any participant who needed psychological support; those who needed medical care or advanced psychological support were referred to appropriate health professionals. The audio recordings will be destroyed 1 year after the study has been completed.

### Reflexivity

MC is a Ugandan paediatrician with an interest in newborn health who has lived in the study area for more than 15 years. Although from a different ethnic group from the participants in this study, he has experienced similar practices around stillbirths since childhood, these prior experiences may have affected the research process. This influence may have been reduced by the author’s conscious awareness of his own beliefs. He did this research as part of his PhD training. One of the four research assistants was born and grew up in the community interviewed, while the other three were raised mostly from central Uganda. Both MC and the research assistants had had training in qualitative research. The study was conceived, planned, implemented and analysed by a multidisciplinary team of scientists (MC, AN, OOP, KB, DM, ADW, NRO, TT, JR and IMSE) that included paediatricians, public health and global health specialists with vast experience in qualitative research. The multidisciplinary nature of the team may have increased the reflexive awareness during the research processes.

### Theoretical lens

The results were viewed through the symbolic interactionism theoretical framework [15]. We did not design the study to collect data hinged on the tenets of symbolic interactionism. This framework was chosen at data analysis and interpretation stage to be used as theoretical lens to interpret the results of the study. The theory proposes that people behave in a particular way towards ‘symbols’ (that could be situations, objects, phenomena, or events) based on the meanings they attach to them (in this case stillbirths) and formed during social interactions about that entity. It argues that people do not just adopt pre-existing values and norms but that their interpretation of objects/situations are shaped by their ongoing interactions and can change over time. A society is a group of people who view symbols in a similar way with shared, but evolving, meaning [16]. The humans therefore give meaning to these symbols and they express this meaning through language and behaviour [16]. Our current study explored how study participants viewed stillbirths and related practices as symbols with both cultural and social meanings.

## Results

The text below summarises the results of the data collection and analysis and contains explicit descriptions of traditional beliefs and activities around stillborn babies and their parents which some readers may find upsetting. We have made our best efforts to synthesize and present these findings as objectively as possible.

We conducted six FGDs. The characteristics of the participants of the FGDs are shown in Table 1. We also conducted 30 IDIs: 12 with women who had experienced a stillbirth; four with men whose spouses had experienced a stillbirth; two with men whose daughters had experienced a stillbirth, six with healthcare workers (four midwives, two doctors), four with religious leaders, one with a cultural leader, and one with a traditional birth attendant, Table 2.

**Table 1:** Characteristics of participants of focus group discussions

Characteristic	Frequency (*N* = 44)
**Age (years)**	
20–40	19
40–60	22
Above 60	3
**Gender**	
Male	18
Female	26
**Education (years)**	
0 to 7	27
8 to13	15
Above 13	2
**Ethnic group**	
Gisu	32
Other	12
**Religion**	
Protestant	16
Other Christian	14
Moslem	14
**Occupation**	
Unemployed	30
Village Health Team member	7
Other	7
**Number of children**	
1 to 3	12
4 to 6	13
7 and above	19
**History of stillbirth**	
Yes	23
No	21

**Table 2:** Characteristics of participants of in-depth interviews

Characteristic	Frequency (*N* = 30)
**Type of participant**	
Woman with stillbirth	12
Male partner of woman with history of stillbirth	4
Father or mother of woman with history of stillbirth	2
Traditional birth attendant	1
Health workers	6
Religious leaders	4
Cultural leaders	1
**Age (years)**	30
20–29	10
30–45	14
Above 45	6
**Gender**	
Female	18
Male	12
**Education (years)**	
0–7	17
8–13	11
Above 13	2
**Ethnic group**	
Gisu	21
Other	7
**Religion**	
Protestant	17
Other Christian	8
Moslem	5
**Number of children**	
0–4	14
5 and above	16
**History of stillbirth**	
Yes	21
No	9

**Fig. 1:**
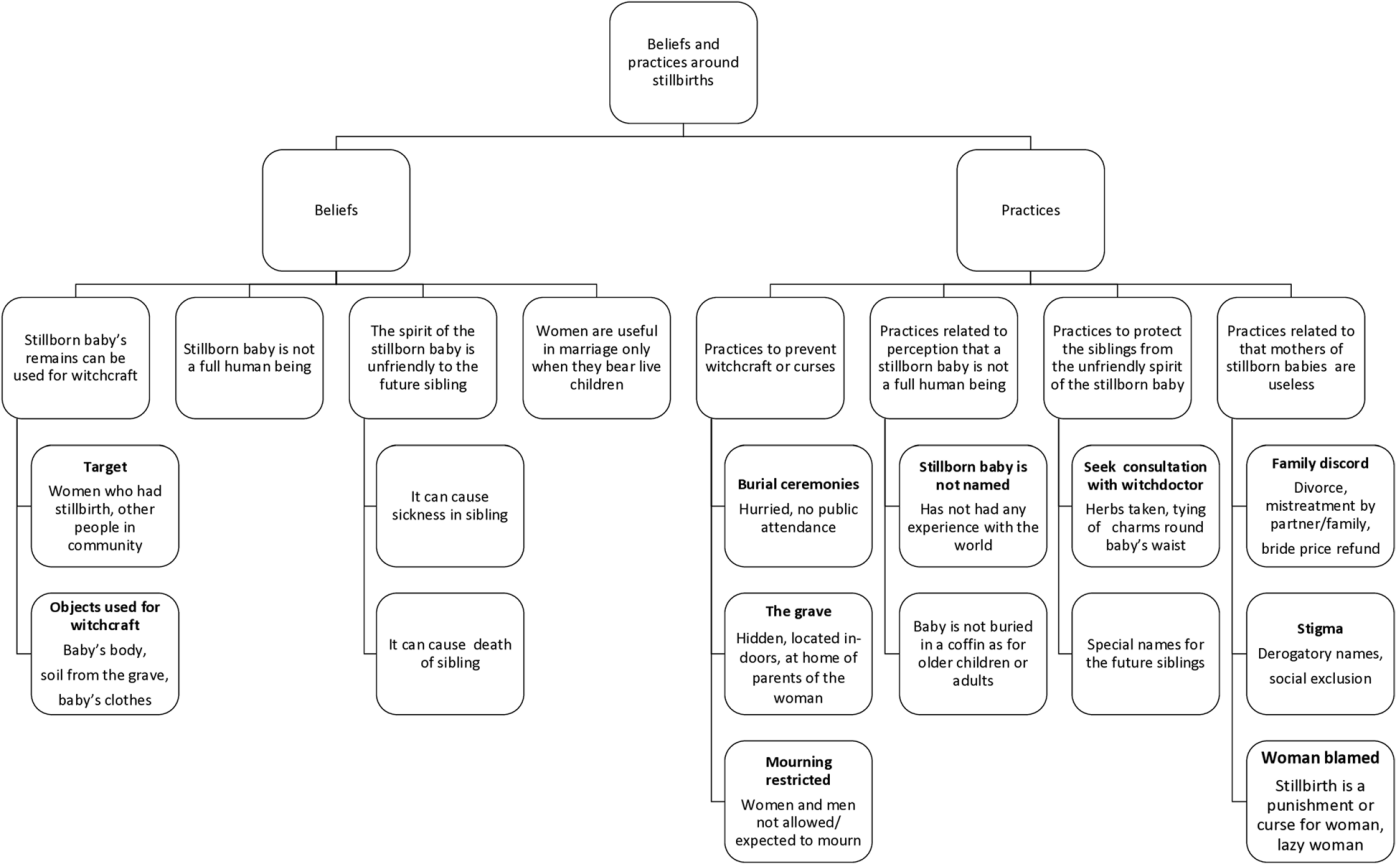
Details of the themes that emerged

The beliefs that emerged include: the stillborn baby’s remains can be used for witchcraft, the stillborn baby is not a full human being, the spirit of the stillborn baby is unfriendly to the future sibling, and women are useful in marriage only when they bear live children. The practices that emerged were related to the beliefs about stillbirths, Fig. 1.

### Beliefs about stillbirths

In addition to being viewed as a loss of a loved one, a stillborn baby was viewed as a symbol with deep spiritual, cultural and social meanings. The meanings attached to it included a ‘weapon of witchcraft’, ‘it is not human’ and ‘it is harmful to future siblings. The soil around the grave and the clothes used to wrap the stillborn baby were also viewed as symbols that could be used for witchcraft. Following exposure to education and the influence of religion and Western culture, some people have developed different meanings around stillborn babies; they view them as a loss of a loved one in the same way the death of an older child or adult is viewed.

### The stillborn baby can be used for witchcraft

It was a common belief in the community that the body parts of any stillborn baby, the soil from the grave from which the baby was buried, or the clothes used to carry the baby could be used for witchcraft. It was said that usually the mother was the target, but the witchcraft could be directed to other people. Most of the participants perceived that witchcraft practitioners (or ‘witch doctors’) had supernatural power to cause adverse experiences to people or release them from such. They therefore had deep fear in it because they believed that a witch doctor could cast a spell on anyone they wanted to. Most respondents believed that any woman who had had a stillbirth could be bewitched and was destined to have recurrent stillbirths in the future. Although the religious leaders believed that witchcraft was being practiced and its supernatural power, they believed that one could not be affected by witchcraft if they prayed to God against it.

The person performing witchcraft was usually someone who did not wish the woman well, for example co-wives (a common cultural practice in this ethnic group). It was perceived by some participants that it was only the body parts of a firstborn baby that could be used for witchcraft and could condemn a mother to recurrent stillbirths. Some women, men and one religious leader perceived that if a woman had had a live birth prior to her stillbirth, they could not be bewitched to have future stillbirths because their womb had already *opened* [given birth to a live baby].

Healthcare workers who had had stillbirths in their health facilities perceived that witchcraft was a very common belief among their patients. Witchcraft was perceived to play a very central role in the culture and the strong belief in it influenced most other practices around stillbirths including immediate and private burial ceremonies, hiding the grave and securing the clothes used to wrap a stillborn infant.

*We have clans (that women get married into) that are full of witchcraft, they can come and get that grave and go and bewitch (the woman) (Participant (P) 4, female, FGD CHWs).*

One healthcare worker from the same community confirmed that she had witnessed a situation where a witch doctor exhumed the body of a stillborn baby to use it for witchcraft although this happened several years ago.

*Our neighbour lost a kid, after losing a kid, the other one who was a witch went and dug pulled the baby from the grave, I don’t know what happened after that because I was still young. (P 04 IDI, female health worker)*

### Women are useful in marriage only when they bear children

Participants perceived that women were married primarily to bear children and were not valued in marriage if they had no living children. The bride price paid was viewed as an investment. This meant that if the woman did not give birth to a live child, she was of no value to the husband.

*When a girl gives birth to a dead child, some men will not want them [in their marriage], they think she has started losing children. They will not want her and might not pay the bride price because she has started losing children. Even the husband’s people will not like her, so your daughter will be there struggling, the husband may not care about her or even his parents do not want her. (P 41, female, FGD older women).*

### A stillborn baby is not a full human being

Several respondents mentioned that a stillborn baby was not regarded as a human being even when the baby was born at term. Most of them mentioned that a stillborn baby was considered a miscarriage, not an infant. Although this was a common belief, a few participants believed the stillborn baby should be considered similarly to the death of an older child or an adult. The distinction commonly came down to whether there was an initial cry of the baby before the death.

*The baby who has been born even if at 7 months [gestation] and it cries, it is considered that the child has died because it cried. They will even pray for the child during burial because it cried. One did not cry after birth is said to be a miscarriage*. *(P 03, FGD CHW)*

### The stillborn baby is unfriendly to the next sibling

Many men, women, and CHWs perceived that the stillborn spirit remained around the grave of the stillborn baby for years and it was usually harmful to the subsequent sibling. It could cause that sibling to fall ill frequently. The stillborn baby’s spirit could also cause the sibling to die by *calling him/her into the world of the dead*.

*The spirit of the stillborn baby can come to the newborn baby and he/she gets convulsions or be called to the dead. (P 01, IDI religious leader).*

### Practices surrounding stillbirths

#### Practices to prevent witchcraft or curses

The participants said it was a common practice to keep the stillbirth a secret because of the fear that if people knew where the baby was buried, the body could be exhumed by witches and used for witchcraft.

*This baby who was already dead before birth, culturally people should not know where is buried because they say that they can use it as witchcraft. That baby, they do not mourn loudly, they do not pray for the baby*. *(P 03, FGD CHW)*

Participants mentioned that the burial of a stillborn baby was different. It was usual practice for such burials to be attended by only close family members. Although a few women who had experienced stillbirths had distant relatives and the community present during the burial, most of them had only close family members present. In contrast, the women, men and CHWs confirmed that burial ceremonies for older children and adults were normally attended by hundreds of people from the neighbouring community. Additionally, it was perceived that there was no need to invite the public for the burial of a stillborn baby since the baby had not formed any attachment with other people in the village. Limiting the number of people attending the burial would prevent the public (among whom are witch doctors) from knowing the site of the grave. The women who had invited the community to attend the burial knew about the *risk* of witchcraft but were not afraid of it because they believed that they were protected by God. In the validation meeting, it was unanimously agreed that the belief and fear of witchcraft was almost universal in this community, even among many religious and educated people.

*My mother took it and buried it. The second one it was also my mother that took it, wrapped it and they went and buried… They showed me where they were buried. My parents said, ‘prayed for them as your mother and father’. …But they buried them at night, when there is no one watching. They buried them, only two people [participated in the burial]. (P 14, IDI woman with history of stillbirth)*

Unlike other deaths, where people could wait for up to three days for other relatives to join them before the burial took place, the burial for a stillbirth was done immediately after the birth. Almost all the women who had had a stillborn baby had buried their baby within 24 h of the birth. Even the women who had invited their community to attend the burial had held the ceremony within one day. Some CHWs, women and healthcare workers confirmed that even when the stillbirth occurred at night, most burials took place before daybreak.

*[The burial process is…] very different actually as soon as you remove [deliver] the baby, they are supposed to rush, they don’t even give you time to do any investigations, they are supposed to rush with the body and they go and bury immediately*. *It’s not supposed to sleep in the house. It’s only a mature baby who should, I mean a baby who came out maybe died after a month or so, but a stillbirth? no, it’s not supposed to stay in the house. That’s why they rush and go and dig [the grave]. (P 05, in depth interview, health worker)*

Most participants mentioned that to avoid access by witches, the baby would be buried in a place where no one should see it. If the baby was buried in the family graveyard outdoors, they may have to exhume it later and transfer it to a secret place, normally far away from the home, for example in the forest or bushes so that no one can access it. To make it easier to exhume the body in the future, unlike other graves where concrete is used, for a stillborn baby, they only covered the grave with soil. Some educated and religious people did not hide the grave of the stillborn baby because they either felt protected by God or they felt that witchcraft did not have the supernatural power to cause harm to anyone’s life. The validation meeting emphasized that the practice of hiding graves of stillborn babies was a very common, even among the educated and the religious people.

*For me I am a born-again Christian. When a woman gives birth to a baby already dead in the womb, and she is in the hospital, and the woman has remained there, we do not hurry to bury at night. We wait until morning and the prayer is very short. In addition, only family members are around. (P 27, FGD young women)*

Although not commonly practiced these days, a stillborn baby was supposed to buried inside the family’s house or on the veranda of the house. Only one male spouse whose partner had had a stillbirth confirmed that his stillborn baby was buried inside the house. In addition, one FGD participant also confirmed they had witnessed a stillborn baby being buried inside the house. None of the participants of the validation meeting had heard about or witnessed a stillborn baby being buried inside the house. They mentioned that it is not a practice known among the Gisu tribe.

*Actually, it’s true the Gishu have that custom of burying in the house. Mine too was buried in the house. Maybe I should say that Gishu people are the best at witchcraft… So, in the house it is a bit safer. In addition, when they bury, even when other people come to console you, they should not know where you buried. (P 16, IDI male partner for woman with history of stillbirth).*

Unlike the graves for older children and adults, which are raised above the ground and therefore conspicuous, the grave of a stillborn baby is made level with the ground. The surrounding ground is dug so that the whole ground looks uniform, so that no-one can tell where the grave is. It is only a few people who, because of religious influences, keep the grave heaped because they do not believe in witchcraft. This practice was also recognised to be common at the validation meeting.

*After burial they spread the soil like in the garden. They just dig and dig. It becomes a garden, and you will not know the location of the grave but the close relatives know. (P 03, FGD CHW).*

The majority of participants mentioned that stillborn babies were buried at the matrimonial home. In some families, the burial was said to take place at the maiden home of the woman. Burial at the maiden home was done to avoid the partner and his relatives from accessing the body to use it to bewitch the woman especially if she decided to leave the marriage. The participants and the people who attended the validation meeting perceived that this practice was more likely to occur if the relatives perceived that the marriage may break up after the stillbirth. They perceived that women for whom the bride price had not yet been paid were more likely to have their marriage breakup. If bride price had been paid, the family of the male spouse may be reluctant to break the marriage because of the *investment they have put in the woman*. Those who had lost their firstborn to stillbirth were also more likely to have dissolution of marriage because of the perceived higher likelihood of future stillbirths.

*Where I come from, there is a woman who had twins and they died, they buried at her husbands’ home in the absence of the people from her side. Two days passed and the people from her side [her relatives] came and said they had done a very bad thing; they asked for where they had buried the babies because they needed to exhume the bodies and transfer to her parents’ home. They tried as much as they could, exhumed the bodies and took them because they thought that the girl was going to be bewitched. (P 37, FGD older men)*

Rituals were also perceived to be quite common after a stillbirth. They were done when the suspected cause of stillbirth was witchcraft or if it was perceived that the spirits were unhappy with the woman or her partner. Sometimes, the woman had to go to her parents to be cleansed before she came back to her husband. The rituals involved chewing certain herbs or performing a cleansing ceremony, which was usually conducted where the roads crossed near the woman’s residence. The woman and her partner were asked to eat roasted meat mixed with herbs, drink local brew and sing and chant, thus inviting the angry spirits to settle and not harm the couple and their future children. Although still practised, the validation meeting participants mentioned that it was not as common as it used to be. Some people did not participate in these rituals because of religious beliefs or the influence of education. Among the women who had had stillbirths, some reported participating in these rituals, others reported declining to participate in them, while others were not approached or did not know about the rituals.

*That [cleansing rituals] will only happen if people are saying that she [mother] met with something or a bad spirit along the way while she was still pregnant, and those bad spirits entered her body and caused her to have a stillbirth. They will try so hard to find a traditional healer where they will take her and she will be taken to a place where roads cross. There she will be washed with some medicine to cleanse her of the evil spirits that she met*. *(P 31, FGD older women)*

Many of the women who lost their babies were not given time to mourn. Most participants said this occurred because the burial was done before the mother was discharged from hospital – when she returned home, she found that the burial had already taken place. Health workers also perceived that women were commonly restricted from mourning the death of their stillborn baby and rarely got a chance to hold their baby before it was taken for burial. It was common for the baby to be taken home from hospital even before the women had learned that she had had a stillbirth. Many healthcare workers had been told by the friends and relatives of the women not to break the bad news to the woman because they feared that the woman’s body was fragile after childbirth. Knowing about the death of her baby was perceived to lead to an excessive emotional reaction causing deterioration of her own health.

*No, … [I was not given the baby to hold] … They took the body immediately. I was discharged that morning when they had finished to bury. (P 06, woman with history of stillbirth)*

Similar to most Ugandan communities, the Gisu usually mourn by wailing when they lose a loved one, but it was reported that people were not supposed to cry out loud after a stillbirth. The reasons given for this practice were inconsistent. Some believed that there was no need for wailing because the stillborn baby was not considered human. Others reported that wailing brought curses to the family of a stillborn baby. In the validation meeting, it was pointed out that the restriction of mourning occurred among some people, but most people were allowed to mourn liberally. It was said that the main reason for restricted mourning is to avoid curses of recurrent stillbirth against the woman.

*... the only thing that looks like the cultural practice is that they say that you should not cry. They do not want to see anyone who is crying. (P 05, FGD CHW)*

Men were supposed to be strong. A man who wailed overtly was viewed as being a weak man. Men therefore concealed their emotions in times of grief. Some reported crying in private and appearing “emotionally strong” in public. Some male respondents, however, believed that it was normal for a man to mourn the way they feel like, including wailing. Some men who had experienced stillbirths stated that they wept in public when they lost their babies. Although most healthcare workers perceived that men should mourn the loss of the baby just like any other person did, a few stated that they did not expect a man to cry like a woman. They mentioned that instead, the man should be the one to counsel and re-assure the woman. The healthcare workers reported that although most men they had encountered had not broken down emotionally, they had witnessed a few men wailing when their partners had a stillbirth.

*I hid myself and cried. My wife, from January up to the July was pregnant carrying all the baby`s weight. It cost me money up to 307,000 shillings (approx. 100 United States Dollars [USD]), now all that has gone for nothing. She had bought baby bed sheets to prepare for the new baby. Eh! That is so painful! I cried! I cried! It made me sorrowful. Yes, you do not cry in public, in the public you have to be strong because you have to welcome the visitors who have come to mourn. (P 15, FGD young men))*

Some women reported that men did not give great significance to a stillborn baby if they had already had other children from other women who were alive.

*“For them, some men are not concerned about mourning a stillbirth. They drink alcohol and say that: ‘ah, me I have produced [meaning I have many children], I have produced, now even if I lose, I don’t care much’. They do not care. Generally, they do not care. So, they do not take a stillbirth as something important.” (P 47, FGD older women)*

#### Practices associated with the women being useful only when they bear live children

Men wanted only women who produced live children, so they often sent the woman back to her parents and demanded their bride price back if they had a stillbirth. The man then sought to *remove* the woman from the marriage (‘divorce’ in Western language) and marry another woman. This was a source of great distress to affected women because marriage is highly valued in this society; married women are respected while those who are unmarried feel undignified and *‘incomplete’*. In the validation meeting, it was also confirmed that divorce or family conflict was common after a stillbirth but even more common back in time.

Often the conflict was not just between the woman and the man, but also involved their families. The involvement of the family members was so strong that even when the partners decided to persist with the marriage, the male spouse’s family (usually the mother, father or siblings) would demand a separation. Although previously, most male partners would submit to these demands, some men now decide to continue with their marriage without their families’ *‘blessings’* these days. When the marriage is upheld, the woman may face serious mistreatment by the partner’s relatives bringing her psychological distress and this may lead to her leaving the marriage anyway. Some of the men interviewed confirmed that their partners left their marriage after the stillbirth.

*They don’t like her, especially her mother-in-law or her sister-in-law or sometimes the father-in-law or even her husband too because he looks at her as a waste of his time. He has invested money in her. Like those who pay education fees for their wives while they are still in school. They say, ‘I paid my money, I thought she was going to give birth, now this is an omen’. (P 03, IDI religious leader)*

Following a stillbirth, the families of the married couple were often locked in conflict. Participants said that this occurs for several reasons: the family of the woman may feel their daughter is being mistreated by the partner and his family or they may be unwilling or unable to pay back the bride price in case of separation of the couple, the family of the partner may also blame the woman or her family for the stillbirth. If she left the marriage and the partner or his relatives were not happy about it, they could use the body parts of the buried stillborn infant to bewitch the woman so that she would not give birth to live children in her next marriage.

*‘They are the ones [relatives of the man] who said “we don’t want. You should at least get another wife. We are tired, we can’t bury the dead people to feel here where we are supposed to be planting our maize.” Those relatives of my husband, they talked many words, they abused my parents, they did everything but my husband cared for me. The ones from my side, for them they had no problem. Of course, my parents, for example my father were only lamenting that they had accused him of bewitching his daughter to have stillbirths’ (P 08, IDI woman with history of stillbirth).*

Some women were blamed for losing the baby. They were perceived to have curses that caused them to have stillbirths. Some people believed that these curses were brought upon the women because they did something wrong, or something that was insulting or demeaning to her parents or other elders in the family before she got married. Some people believed it was because she decided to marry a man whom her relatives did not approve of, whilst others perceived that the woman was too lazy or too fat to push the baby out.

*Sometimes families (relatives of the male partner) develop hatred. They tell the man to get another woman because they are tired of seeing sweet potato heaps at home [referring to graves of the stillborn children] from a cursed woman. …they laugh at that mother a lot. We as the owners of the wife, the clan especially your father and mother will turn against you saying “your wife fears pushing babies, she cannot manage childbirth. Look for another woman”. Many other things like that are done or said. (P 16, FGD young men)*

Several women, especially those who have had several stillbirths, experienced stigma. These women confirmed that the family of their partner and their community mocked them. The partners of these women also confirmed that their partners faced stigma. Sometimes the stigma ‘*spilled’* over to the male partners who reported being mocked by friends and being blamed for the occurrence of a stillbirth in their family. This caused profound psychological torture to them and would make them contemplate ending their marriage.

*My wife would go to the well and come back crying. She might go to the party, a person who was seen pregnant... When she goes to church other people mock her. (P 38, FGD older men)*

Despite stigma being common, several women also reported a supportive environment. Some community members would encourage her to be strong despite the stigma they faced from the community. Some male spouses were reported to be supportive to their female partners.

*That whole time, my husband did not have any problem with me, he cared for me. He said… Ok for him said, “don’t worry, we shall get another baby”. “Be patient, don’t cry because if you cry, God may get angered. Just be patient”. (P 06, woman with history of stillbirth)*

Sometimes women who have had stillbirths were labelled with derogatory names. This would usually be initiated by the immediate family (relatives of the husband) and the whole community eventually follow. They were referred to as *‘the one who gives birth to dead things.* This would make the women frustrated and lose self-esteem. In community or family meetings, these women were not allowed to speak, and their opinions did not matter because *‘Nothing sensible comes from a woman who gives birth to soil’*. In this community, children are expected to help adults in domestic chores such as fetching water and being sent to buy some domestic items such as salt, sugar and soap from nearby retail shops. Usually, one would also engage the neighbour’s children for these activities. However, women in the neighbourhood would discourage their children from helping a woman who had had a stillbirth.

*‘Sometimes – that woman – she loses her marriage. Sometimes either the family or the village people nickname her “Kifisha” [one who has a habit of giving birth to dead babies]. Because every child you give birth to dies. Everywhere you pass they call her “kifisha”. You get a name which is not your original name. (P 03, FGD CHW)*

Some participants reported that some men, influenced by religion or education were understanding and therefore offered support to their partners. They did not blame their partners for the death of their baby. They mentioned that in some religions, couples were taught that the primary reason for marriage is for companionship and that children are an additional blessing in marriage. If they did not have children, marriage was encouraged to continue as they believe in ‘*God to provide the children’*.

*… but if the husband is good, he can chase away his own family parents (who advise him to divorce and marry another woman). Sometimes they (the couple) go and rent somewhere else (away from the relatives) … (P 04, Religious leader)*

#### Practices associated with perception that the baby is not a full human being

Stillborn babies were not given names for several reasons: it was perceived that it was not important to name them because they were not going to live to be called by that name; they were not baptised; and they had not had *“experience with the world”*. It was perceived that the naming of the baby is supposed to be several days after birth, so since the baby died before birth, they should not be named.

*The baby is already dead so why would they name it? They are going to bury it so what would be the purpose of giving them a name if they're not going to be called by that name. So, they'll just bury that one. However, for those who were born alive and lived for some time, they name them, and some might have been baptised already. Like for us the Anglicans they might have already baptised that child therefore the priests would come and pray for it during the funeral. For the stillbirth, they just bury, they do not even invite the church except now for some of us who are born again, we may invite a spiritual leader to stand with us during that time. (P 45, FGD older women)*

Whereas in local burials, the deceased body is usually placed in a coffin, no coffin was generally used for stillborn babies as the baby was not regarded as ‘a real child’. At the validation meeting however, the reason for this practice was to allow the remains to *mix with the rest of the soil* in a short time so that the grave is not traceable.

*What I know with them, they just go dig a hole, get a piece of white cloth, wrap, go and then put some sort of like sticks then that’s the end of it. (P 18, IDI father to woman with stillbirth)*

#### Practices to protect the next sibling from the unfriendly spirit

To protect the life of the next sibling from the haunting spirit of the stillborn baby, the body had to be exhumed if it was buried near the home and the remains taken to a place far away so that the new baby was safe. Furthermore, women sought the services of the traditional healers to protect the subsequent sibling*.*

*Those in the world [unreligious people] will go to the shrines for help. They will try some medicine in a piece of “cloth” and tie it around the baby's wrist as a form of protection so that they don't die like the previous ones. (P 19, FGD young men)*

The children born after a stillbirth were given special names, especially if the woman had lost several children. Some participants mentioned that the names were given because it was believed that, when given this name, the child would not die due to the haunting spirit of their stillborn sibling. Others said that they just served to identify that the child was born after a stillbirth. Several women mentioned that some people no longer followed this tradition but instead chose any name they would like to give to their child.

*When one has lost many children, they might name the children born later depending on the circumstances such as ‘Gudoi’ [from the word ‘lidoi’ meaning ‘soil’], Kutaka [‘taka’ meaning ‘the ground’], kuloba [from the word ‘liloba’ meaning ‘soil or ground’], Nambafu [from the word ‘mufu or bafu’ meaning ‘the dead’]. Previously, they would use the name Kuloba to mean that the child he followed was eaten up by the soil. The elders back then thought that if they named Kuloba then the child would not die like the others did. What I know is that nowadays, people name children depending on the joy they have got out of having the child. (P 37, older men).*

At the validation meeting, the practices of special naming or seeking services of traditional healers were said to still occur but were perceived to be less common than several years ago because of the influence of religion and education.

## Discussion

The results of our study reveal many common beliefs around stillbirths in Eastern Uganda. These include the belief that a stillborn baby can be used for witchcraft, that women are married with the main aim of bearing children, that stillborn babies are not considered human beings and that a stillborn baby’s spirit can be unfriendly to the next siblings. The belief in witchcraft is so central in this community that many practices exist to prevent witch doctors from accessing the body of the stillborn baby, their clothes or the soil from their grave in order to harm the mother or others. This results in rapid, hidden burials, and restricted mourning. Blaming the mother for the stillbirth of her baby, beliefs in the curse of future pregnancies, stigmatisation and bullying led to psychological harm, marriage break-up and social exclusion of these women.

These results reflect how this community in Eastern Uganda attaches extraordinary meanings to ordinary objects through shared beliefs. In addition to being a tragedy, to some people, a stillborn baby is feared as a source of danger through witchcraft, a result of a curse, a punishment to the woman for bad behaviour or a harmful spirit to future siblings. Witchcraft symbolised supernatural influence leading to fear and this belief shaped most practices surrounding stillbirths. Ordinary objects such as soil from the grave and the clothes and body of the stillborn baby were viewed to have supernatural power to influence one’s life when used in witchcraft and so should be hidden from the public, among whom are witch doctors. The private, often hurried burials represented the symbolic importance of reducing the interaction with the stillborn baby from the community among which are witch doctors. Marriage symbolised an institution whose aim is to bear children to ensure continuity of the family line. Bride price symbolised a tool to validate marriage and cement woman’s status in a marriage. The woman was also an object of symbolic interactionism where her success in marriage largely hinged on the success or failure in childbirth. The social exclusion and stigma suffered by women who had stillbirths resembled the treatment of a someone who is cursed or facing punishment because of bad behaviour.

Our findings showing a strong belief about witchcraft are similar to a study in Uganda and Kenya that found that the community believe that the body of a stillborn baby can be used for witchcraft [5]. The finding that burials are done soon after birth by only close family members are similar to what other studies have found in Ethiopia [7]. Similar to our study, they also found that in the studied Ethiopian community, stillbirths are hidden from the community and in some instances, even the mother of the stillborn baby is not supposed to know where the baby was buried. This practice is likely to affect the accuracy of documenting the burden of stillbirths in these communities and the interventions designed to reduce stillbirths needs to be customised to meet cultural considerations. It is therefore necessary that such public health programmes design programmes in consultation with the community.

Stillborn babies were not considered to be human and were therefore not named. This finding is similar to the Ethiopian study that also found that stillborn babies were not considered human and that it did not carry any meaning to feel sad or mourn their death. After the burial of the baby, “the stillbirths are forgotten” and no one should discuss about it [7]. The study in Uganda and Kenya also found that stillborn babies are not considered human and hence are not named [5]. This has direct implications for the recording of stillborn infants.

Our study found women to be devalued if they do not have an alive child. When they experience stillbirth, they may be blamed and mistreated and sometimes may be forced to leave their marriage, although this was described as being less common today than previously. This finding is again similar to the Ethiopian study that found women were sometimes blamed for the stillbirth and sometimes forced to divorce [7]. In addition, women were treated or talked about as assets that could be sent back to her family and the bride price could be claimed back. The language used by the participants such as *‘we the owners of the wives’* reflects that a woman is sometimes viewed as an asset with no autonomy. Although this may be true for some women, several women had a degree of autonomy, for example choosing whom to marry, although sometimes they faced rejection from the family if they did not approve of the marriage. A previous study in Uganda found that bride price payment is perceived to indicate that the woman has been ‘bought’ to the man’s family leading to unequal gender power relations, reduced decision making roles and independence of women [17].

Grief related to lost investment opportunities were exacerbated by poverty and despair related to their prior investment and expenditures with the transport and services. Previous studies in Uganda, Tanzania and Malawi found that catastrophic health expenditures are common [18].

In this study, we found that people, especially the women, were restricted from mourning. This finding is similar to studies previously done in Ghana, Ethiopia, Kenya and Uganda that found that women are dissuaded from crying [5, 7, 19]. They also did not get a chance to hold their babies or attend burials because it was against the customs [5, 7]. The reasons for the restricted mourning in our study were similar to these studies; either because the stillborn baby was not regarded as human or the perception that one brings bad misfortune such as future stillbirths to the same family if they mourn overtly. Men were expected to appear strong and not wail like women. Instead, they should console the women. This placed a psychological strain on the men and this was further compounded by the financial losses the men had to bear.

The perceived unfriendliness of the spirit of the stillborn baby was perceived to cause illnesses or death of the next siblings. Some studies have reported similar findings in Taiwan where it was perceived that the spirit of the stillborn child could cause misfortune to the family if the funeral affairs were mishandled [20]. When siblings of these stillborn babies fell sick, instead of seeking medical care, the parents would seek the intervention of a traditional healer. This may lead to delays in accessing the right treatment for the condition of the child leading to adverse health outcomes including lifelong complication or death.

Despite widespread access to primary education in rural Ugandan communities today, many societies still hold strong traditional beliefs about medical conditions and death. In this study, we found that although cultural beliefs and practices were common, some people no longer practice them. Previous studies in Uganda have also found similar results [21]. These communities are shifting from cultural practices to more biomedical explanations for stillbirths due to the influence of education, religion and so-called “western culture” dominated by higher education and biomedical explanations. The shift to biomedical explanations for causes of stillbirths also influences the practices around stillbirths. For example, in Uganda, because of this shift, some communities consider stillbirths to be similar to death of an older child or adult. In these communities, burial practices and mourning are similar to death of older children and adults. The community we studied had cultural beliefs and practices more widely practiced than communities that have demonstrated a biomedical shift.

Although the descriptions may be difficult for many to read, they represent the reality of what many mothers in the world face when stillbirths occur. These beliefs and practices are likely to hinder the implementation of stillbirth policies. This understanding is critical if mothers and fathers who suffer stillbirths are going to be supported and when designing interventions to be used in traditional communities. Interventions that might be welcomed in a modern setting such as memory boxes or memorial plaques could easily be seen as dangerous in the context of a widespread belief in witchcraft. This, once again, serves as a reminder that global health research and interventions should never be conducted without a full understanding of the local culture and only in partnership with local communities.

This study had weaknesses and strengths. The study depended on the subjective responses from the participants and researcher’s interpretation. As such, there is always room for response and interpretation bias which may affect trustworthiness of the findings. In order to mitigate this potential weakness, most of the findings from the first larger phase of data collection and analysis was later presented in a validation meeting with a new set of participants from the same area confirming the findings. The study involved many community representatives including women, men, community, cultural and religious leaders and healthcare workers so that the information could be triangulated and verified. This improves the credibility of our findings. It was also conducted in the local languages with locally trusted healthcare workers, meaning that the respondents were likely to have been open about their beliefs and practices. Although the interviewers were healthcare workers, the strong emphasis on witchcraft may signal honesty and trust in the informants’ responses. However, the study was from a small area of rural Uganda and may not represent belief systems in other communities either inside or outside of Uganda.

## Conclusion

In conclusion, traditional beliefs still strongly influence the perception and practices around stillbirths among the studied community in Eastern Uganda. The strong beliefs in witchcraft against the mother influenced most practices surrounding stillbirths. These practices mostly entailed secrecy and hiding of stillbirths. These cultural practices in Uganda are likely to contribute to underreporting of stillbirths. Efforts to improve accuracy of stillbirth recording needs to take this into account.

The belief that a stillborn baby was not human, the spirit of the stillborn baby could harm their new sibling and that women are only useful in marriage when they bear children also led to several practices surrounding stillbirths.

We recommend that culturally sensitive interventions should be incorporated in stillbirth policies including, but not restricted to awareness raising, education and reproductive health services. These results will be helpful in shaping support for women in communities where stillbirths occur. We also recommend that sensitization is done among these communities to emphasise the biomedical explanations of stillbirths. We recommend that further research is done to document the extent, nature and diversity of beliefs and practices around stillbirths in different communities in sub-Saharan Africa.

## Data Availability

The dataset used and/or analysed during the current study are available from the corresponding author on reasonable request.

## Abbreviations

CHWs: Community health workers
FGDs: Focus group discussions
IDIs: In-depth interviews
LMICs: Low- and middle-income countries
MRRH: Mbale Regional Referral Hospital
SSA: Sub–Saharan Africa
USD: United States Dollars
